# *N*-Cyclo­hexyl­cyclo­hexa­naminium chloride

**DOI:** 10.1107/S1600536811000857

**Published:** 2011-01-12

**Authors:** Mehrdad Pourayoubi, Monireh Negari, Marek Nečas

**Affiliations:** a Department of Chemistry, Ferdowsi University of Mashhad, Mashhad 91779, Iran; b Department of Chemistry, Faculty of Science, Masaryk University, Kotlarska 2, Brno CZ-61137, Czech Republic

## Abstract

In the title salt, C_12_H_24_N^+^·Cl^−^, both cyclo­hexyl rings adopt chair conformations and the NH_2_ unit is situated in the equatorial position with respect to the rings in the cation. The large C—N—C bond angle [117.99 (14)°] in the cation is a result of linking two bulky cyclo­hexyl rings to the N atom. The aminium H atoms are involved in inter­molecular N—H⋯Cl hydrogen bonds, forming an infinite zigzag chain parallel to the *c* axis. The crystal studied was a racemic twin with a twin fraction of 0.28 (18).

## Related literature

For related structures, see: Gholivand & Pourayoubi (2004 ▶); Pourayoubi & Negari (2010 ▶). 
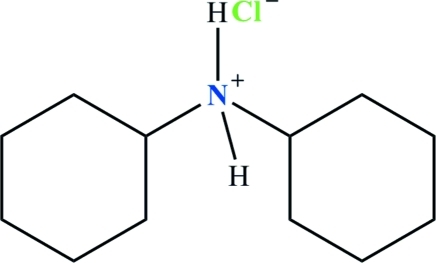


## Experimental

### 

#### Crystal data

C_12_H_24_N^+^·Cl^−^
*M* *_r_* = 217.77
Orthorhombic, 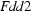

*a* = 40.0268 (19) Å
*b* = 23.1726 (10) Å
*c* = 5.3463 (2) Å
*V* = 4958.8 (4) Å^3^
*Z* = 16
Mo *K*α radiation
μ = 0.27 mm^−1^
*T* = 120 K
0.30 × 0.20 × 0.20 mm

#### Data collection

Oxford Diffraction Xcalibur Sapphire2 diffractometer
Absorption correction: multi-scan (*CrysAlis RED*; Oxford Diffraction, 2009 ▶) *T*_min_ = 0.886, *T*_max_ = 1.000
13243 measured reflections
1219 independent reflections
1096 reflections with *I* > 2σ(*I*)
*R*_int_ = 0.032

#### Refinement

*R*[*F* ^2^ > 2σ(*F* ^2^)] = 0.025
*wR*(*F* ^2^) = 0.057
*S* = 1.00
1219 reflections
136 parameters
1 restraint
H atoms treated by a mixture of independent and constrained refinement
Δρ_max_ = 0.30 e Å^−3^
Δρ_min_ = −0.11 e Å^−3^

### 

Data collection: *CrysAlis CCD* (Oxford Diffraction, 2009 ▶); cell refinement: *CrysAlis RED* (Oxford Diffraction, 2009 ▶); data reduction: *CrysAlis RED*; program(s) used to solve structure: *SHELXS97* (Sheldrick, 2008 ▶); program(s) used to refine structure: *SHELXL97* (Sheldrick, 2008 ▶); molecular graphics: *Mercury* (Macrae *et al.*, 2008 ▶); software used to prepare material for publication: *SHELXL97*.

## Supplementary Material

Crystal structure: contains datablocks I, global. DOI: 10.1107/S1600536811000857/pv2369sup1.cif

Structure factors: contains datablocks I. DOI: 10.1107/S1600536811000857/pv2369Isup2.hkl

Additional supplementary materials:  crystallographic information; 3D view; checkCIF report

## Figures and Tables

**Table 1:** Hydrogen-bond geometry (Å, °)

*D*—H⋯*A*	*D*—H	H⋯*A*	*D*⋯*A*	*D*—H⋯*A*
N1—H1*N*⋯Cl1^i^	0.87 (2)	2.28 (3)	3.157 (3)	178 (2)
N1—H2*N*⋯Cl1	1.03 (3)	2.15 (3)	3.163 (3)	168 (2)

Symmetry code: (i) 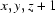.

**Fig. 1.**
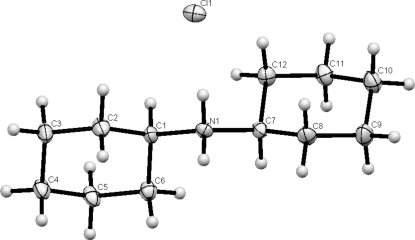
Molecular structure and atom labeling scheme for title compound with displacement ellipsoids at the 50% probability level.

## supplementary crystallographic information

### Comment

The crystal structure of C_10_H_16_N^+^.Cl^-^ was reported recently (Pourayoubi & Negari, 2010). In continuation of our investigations, we report in this paper the preparation and crystal structure of the title salt.

In the title salt (Fig. 1), the cyclohexyl groups adopt chair conformations and the NH_2_ unit is situated in the equatorial position with respect to the rings. The C–N bond lengths of 1.502 (2) and 1.516 (2) Å are in agreement with the corresponding bond lengths reported in closely related compounds (Pourayoubi & Negari, 2010; Gholivand & Pourayoubi, 2004).

A large C1—N1—C7 bond angle (of 117.99 (14)°) in the cation, (C_6_H_11_)_2_NH_2_^+^, is a result of the two bulky C_6_H_11_ groups linked to the N atom. The nitrogen bound H atoms are involving in intermolecular N—H···Cl hydrogen bonds (N···Cl = 3.157 (3) and 3.163 (3) Å) to form an infinite zigzag chain parallel to the *c* axis.

### Experimental

The title compound is a by-product of the preparation of P(O)[OC_6_H_5_][N(C_6_H_11_)_2_]_2_ [from the reaction between P(O)(OC_6_H_5_)Cl_2_ and NH(C_6_H_11_)_2_, in 1:4 mole ratio]. Single crystals were obtained from a solution of ethanol at room temperature.

### Refinement

Carbon bound hydrogen atoms were included in the refinement at geometrically idealized positions with distances C—H = 0.99 and 1.00 Å for methylene and methyne type H-atoms and their *U*_iso_ were set to 1.2*U*_eq_ times of their parent atoms. Nitrogen bound hydrogen atoms were located in a difference Fourier map and refined isotropically. In final refinement cycles, racemic twinning was taken into account, giving a twin fraction of 0.28 (18); Friedel pairs (967) were merged.

### Crystal data

**Table:**

C_12_H_24_N^+^·Cl^−^	*F*(000) = 1920
*M**_r_* = 217.77	*D*_x_ = 1.167 Mg m^−^^3^
Orthorhombic, *F**d**d*2	Mo *K*α radiation, λ = 0.71073 Å
Hall symbol: F 2 -2d	Cell parameters from 6909 reflections
*a* = 40.0268 (19) Å	θ = 2.8–27.3°
*b* = 23.1726 (10) Å	µ = 0.27 mm^−^^1^
*c* = 5.3463 (2) Å	*T* = 120 K
*V* = 4958.8 (4) Å^3^	Block, colorless
*Z* = 16	0.30 × 0.20 × 0.20 mm

### Data collection

**Table:**

Oxford Diffraction Xcalibur Sapphire2 diffractometer	1219 independent reflections
Radiation source: Enhance (Mo) X-ray Source	1096 reflections with *I* > 2σ(*I*)
graphite	*R*_int_ = 0.032
Detector resolution: 8.4353 pixels mm^-1^	θ_max_ = 25.0°, θ_min_ = 3.5°
ω scans	*h* = −25→47
Absorption correction: multi-scan (*CrysAlis RED*; Oxford Diffraction, 2009)	*k* = −26→27
*T*_min_ = 0.886, *T*_max_ = 1.000	*l* = −6→6
13243 measured reflections	

### Refinement

**Table:**

Refinement on *F*^2^	Primary atom site location: structure-invariant direct methods
Least-squares matrix: full	Secondary atom site location: difference Fourier map
*R*[*F*^2^ > 2σ(*F*^2^)] = 0.025	Hydrogen site location: inferred from neighbouring sites
*wR*(*F*^2^) = 0.057	H atoms treated by a mixture of independent and constrained refinement
*S* = 1.00	*w* = 1/[σ^2^(*F*_o_^2^) + (0.0378*P*)^2^] where *P* = (*F*_o_^2^ + 2*F*_c_^2^)/3
1219 reflections	(Δ/σ)_max_ < 0.001
136 parameters	Δρ_max_ = 0.30 e Å^−^^3^
1 restraint	Δρ_min_ = −0.11 e Å^−^^3^

### Special details

**Table:**

Geometry. All e.s.d.'s (except the e.s.d. in the dihedral angle between two l.s. planes) are estimated using the full covariance matrix. The cell e.s.d.'s are taken into account individually in the estimation of e.s.d.'s in distances, angles and torsion angles; correlations between e.s.d.'s in cell parameters are only used when they are defined by crystal symmetry. An approximate (isotropic) treatment of cell e.s.d.'s is used for estimating e.s.d.'s involving l.s. planes.
Refinement. Refinement of *F*^2^ against ALL reflections. The weighted *R*-factor *wR* and goodness of fit *S* are based on *F*^2^, conventional *R*-factors *R* are based on *F*, with *F* set to zero for negative *F*^2^. The threshold expression of *F*^2^ > σ(*F*^2^) is used only for calculating *R*-factors(gt) *etc*. and is not relevant to the choice of reflections for refinement. *R*-factors based on *F*^2^ are statistically about twice as large as those based on *F*, and *R*- factors based on ALL data will be even larger.

### Fractional atomic coordinates and isotropic or equivalent isotropic displacement parameters (Å^2^)

**Table:**

	*x*	*y*	*z*	*U*_iso_*/*U*_eq_	
Cl1	0.100648 (13)	0.19484 (2)	0.28104 (17)	0.02112 (15)	
N1	0.10617 (4)	0.26694 (6)	0.7818 (5)	0.0166 (4)	
C1	0.14111 (5)	0.29218 (8)	0.7797 (5)	0.0161 (4)	
H1B	0.1433	0.3186	0.6328	0.019*	
C2	0.16565 (5)	0.24213 (9)	0.7477 (5)	0.0228 (5)	
H2A	0.1611	0.2221	0.5877	0.027*	
H2B	0.1625	0.2140	0.8852	0.027*	
C3	0.20173 (5)	0.26402 (10)	0.7495 (5)	0.0258 (5)	
H3A	0.2172	0.2309	0.7360	0.031*	
H3B	0.2054	0.2894	0.6032	0.031*	
C4	0.20916 (5)	0.29750 (9)	0.9898 (5)	0.0237 (6)	
H4A	0.2074	0.2713	1.1354	0.028*	
H4B	0.2322	0.3128	0.9837	0.028*	
C5	0.18455 (5)	0.34732 (8)	1.0204 (6)	0.0227 (5)	
H5A	0.1879	0.3753	0.8828	0.027*	
H5B	0.1891	0.3674	1.1802	0.027*	
C6	0.14823 (5)	0.32636 (8)	1.0181 (6)	0.0207 (5)	
H6A	0.1441	0.3016	1.1660	0.025*	
H6B	0.1330	0.3599	1.0276	0.025*	
C7	0.07713 (5)	0.30801 (8)	0.7845 (6)	0.0169 (4)	
H7A	0.0804	0.3363	0.9238	0.020*	
C8	0.04529 (5)	0.27365 (9)	0.8342 (5)	0.0219 (5)	
H8A	0.0471	0.2538	0.9977	0.026*	
H8B	0.0425	0.2439	0.7031	0.026*	
C9	0.01485 (6)	0.31357 (11)	0.8354 (5)	0.0277 (6)	
H9A	−0.0056	0.2903	0.8594	0.033*	
H9B	0.0166	0.3408	0.9774	0.033*	
C10	0.01206 (6)	0.34760 (10)	0.5914 (5)	0.0262 (6)	
H10A	−0.0068	0.3751	0.6034	0.031*	
H10B	0.0074	0.3207	0.4518	0.031*	
C11	0.04427 (5)	0.38076 (8)	0.5367 (5)	0.0224 (5)	
H11A	0.0473	0.4112	0.6644	0.027*	
H11B	0.0424	0.3998	0.3715	0.027*	
C12	0.07490 (5)	0.34125 (8)	0.5370 (5)	0.0196 (5)	
H12A	0.0954	0.3646	0.5138	0.024*	
H12B	0.0733	0.3136	0.3962	0.024*	
H1N	0.1053 (6)	0.2468 (10)	0.920 (5)	0.012 (7)*	
H2N	0.1029 (6)	0.2391 (12)	0.634 (6)	0.040 (9)*	

### Atomic displacement parameters (Å^2^)

**Table:**

	*U*^11^	*U*^22^	*U*^33^	*U*^12^	*U*^13^	*U*^23^
Cl1	0.0307 (3)	0.0149 (2)	0.0178 (2)	0.0004 (2)	−0.0032 (3)	0.0000 (2)
N1	0.0183 (11)	0.0159 (8)	0.0155 (8)	0.0013 (7)	−0.0006 (9)	0.0034 (10)
C1	0.0125 (10)	0.0191 (10)	0.0169 (10)	−0.0010 (8)	−0.0012 (12)	0.0001 (11)
C2	0.0209 (13)	0.0225 (11)	0.0250 (13)	0.0037 (10)	−0.0036 (11)	−0.0085 (10)
C3	0.0159 (13)	0.0342 (12)	0.0274 (13)	0.0057 (10)	−0.0037 (12)	−0.0105 (12)
C4	0.0171 (13)	0.0275 (12)	0.0265 (13)	−0.0003 (10)	−0.0046 (12)	−0.0067 (10)
C5	0.0184 (13)	0.0217 (10)	0.0280 (12)	−0.0004 (9)	−0.0064 (13)	−0.0072 (13)
C6	0.0191 (12)	0.0195 (10)	0.0235 (11)	0.0022 (10)	−0.0002 (11)	−0.0019 (12)
C7	0.0164 (11)	0.0172 (9)	0.0171 (9)	0.0033 (9)	−0.0024 (13)	−0.0034 (10)
C8	0.0205 (13)	0.0249 (12)	0.0203 (13)	−0.0018 (10)	−0.0001 (10)	0.0081 (9)
C9	0.0170 (13)	0.0374 (14)	0.0288 (16)	−0.0005 (11)	0.0014 (10)	0.0076 (11)
C10	0.0210 (14)	0.0277 (12)	0.0298 (15)	0.0037 (11)	−0.0044 (11)	0.0041 (10)
C11	0.0209 (13)	0.0184 (10)	0.0281 (12)	0.0008 (9)	−0.0028 (13)	0.0017 (11)
C12	0.0187 (13)	0.0162 (10)	0.0240 (11)	−0.0003 (9)	0.0003 (12)	0.0051 (11)

### Geometric parameters (Å, °)

**Table:**

N1—C7	1.502 (2)	C6—H6A	0.9900
N1—C1	1.516 (2)	C6—H6B	0.9900
N1—H1N	0.87 (2)	C7—C8	1.526 (3)
N1—H2N	1.03 (3)	C7—C12	1.534 (4)
C1—C6	1.527 (4)	C7—H7A	1.0000
C1—C2	1.529 (3)	C8—C9	1.530 (3)
C1—H1B	1.0000	C8—H8A	0.9900
C2—C3	1.531 (3)	C8—H8B	0.9900
C2—H2A	0.9900	C9—C10	1.529 (3)
C2—H2B	0.9900	C9—H9A	0.9900
C3—C4	1.530 (3)	C9—H9B	0.9900
C3—H3A	0.9900	C10—C11	1.529 (3)
C3—H3B	0.9900	C10—H10A	0.9900
C4—C5	1.526 (3)	C10—H10B	0.9900
C4—H4A	0.9900	C11—C12	1.530 (3)
C4—H4B	0.9900	C11—H11A	0.9900
C5—C6	1.533 (3)	C11—H11B	0.9900
C5—H5A	0.9900	C12—H12A	0.9900
C5—H5B	0.9900	C12—H12B	0.9900
			
C7—N1—C1	117.99 (14)	C1—C6—H6B	109.6
C7—N1—H1N	107.4 (15)	C5—C6—H6B	109.6
C1—N1—H1N	104.4 (15)	H6A—C6—H6B	108.1
C7—N1—H2N	107.9 (15)	N1—C7—C8	108.49 (15)
C1—N1—H2N	110.7 (14)	N1—C7—C12	110.8 (2)
H1N—N1—H2N	108.0 (17)	C8—C7—C12	111.32 (19)
N1—C1—C6	111.49 (19)	N1—C7—H7A	108.7
N1—C1—C2	107.50 (15)	C8—C7—H7A	108.7
C6—C1—C2	111.50 (18)	C12—C7—H7A	108.7
N1—C1—H1B	108.8	C7—C8—C9	110.51 (17)
C6—C1—H1B	108.8	C7—C8—H8A	109.5
C2—C1—H1B	108.8	C9—C8—H8A	109.5
C1—C2—C3	110.73 (17)	C7—C8—H8B	109.5
C1—C2—H2A	109.5	C9—C8—H8B	109.5
C3—C2—H2A	109.5	H8A—C8—H8B	108.1
C1—C2—H2B	109.5	C10—C9—C8	111.50 (19)
C3—C2—H2B	109.5	C10—C9—H9A	109.3
H2A—C2—H2B	108.1	C8—C9—H9A	109.3
C4—C3—C2	110.89 (19)	C10—C9—H9B	109.3
C4—C3—H3A	109.5	C8—C9—H9B	109.3
C2—C3—H3A	109.5	H9A—C9—H9B	108.0
C4—C3—H3B	109.5	C9—C10—C11	111.2 (2)
C2—C3—H3B	109.5	C9—C10—H10A	109.4
H3A—C3—H3B	108.0	C11—C10—H10A	109.4
C5—C4—C3	110.37 (19)	C9—C10—H10B	109.4
C5—C4—H4A	109.6	C11—C10—H10B	109.4
C3—C4—H4A	109.6	H10A—C10—H10B	108.0
C5—C4—H4B	109.6	C10—C11—C12	112.01 (16)
C3—C4—H4B	109.6	C10—C11—H11A	109.2
H4A—C4—H4B	108.1	C12—C11—H11A	109.2
C4—C5—C6	111.81 (16)	C10—C11—H11B	109.2
C4—C5—H5A	109.3	C12—C11—H11B	109.2
C6—C5—H5A	109.3	H11A—C11—H11B	107.9
C4—C5—H5B	109.3	C11—C12—C7	110.37 (19)
C6—C5—H5B	109.3	C11—C12—H12A	109.6
H5A—C5—H5B	107.9	C7—C12—H12A	109.6
C1—C6—C5	110.4 (2)	C11—C12—H12B	109.6
C1—C6—H6A	109.6	C7—C12—H12B	109.6
C5—C6—H6A	109.6	H12A—C12—H12B	108.1
			
C7—N1—C1—C6	63.7 (3)	C1—N1—C7—C8	−169.8 (2)
C7—N1—C1—C2	−173.8 (2)	C1—N1—C7—C12	67.7 (3)
N1—C1—C2—C3	−178.8 (2)	N1—C7—C8—C9	−179.26 (19)
C6—C1—C2—C3	−56.3 (3)	C12—C7—C8—C9	−57.1 (2)
C1—C2—C3—C4	56.7 (3)	C7—C8—C9—C10	56.2 (3)
C2—C3—C4—C5	−56.6 (2)	C8—C9—C10—C11	−54.7 (3)
C3—C4—C5—C6	56.5 (3)	C9—C10—C11—C12	54.4 (3)
N1—C1—C6—C5	175.55 (16)	C10—C11—C12—C7	−55.0 (3)
C2—C1—C6—C5	55.4 (2)	N1—C7—C12—C11	177.21 (16)
C4—C5—C6—C1	−55.7 (3)	C8—C7—C12—C11	56.4 (2)

### Hydrogen-bond geometry (Å, °)

**Table:**

*D*—H···*A*	*D*—H	H···*A*	*D*···*A*	*D*—H···*A*
N1—H1N···Cl1^i^	0.87 (2)	2.28 (3)	3.157 (3)	178 (2)
N1—H2N···Cl1	1.03 (3)	2.15 (3)	3.163 (3)	168 (2)

Symmetry codes: (i) *x*, *y*, *z*+1.
